# Inundation counteracts the promoting effect of nitrogen enrichment on soil organic carbon mineralization in a tidal marsh

**DOI:** 10.1016/j.fmre.2024.01.013

**Published:** 2024-02-05

**Authors:** Chuan Tong, Ji Tan, Min Luo, Jiafang Huang, Shuyao Xiao, Baigui Liu, James T. Morris

**Affiliations:** a Key Laboratory of Humid Sub-Tropical Eco-Geographical Processes (Ministry of Education), Fujian Normal University, Fuzhou 350007, China; b School of Geographical Sciences, Fujian Normal University, Fuzhou 350007, China; c College of Environment and Safety Engineering, Fuzhou University, Fuzhou 350108, China; d Belle Baruch Institute for Marine and Coastal Sciences, University of South Carolina, Columbia 29208, USA

**Keywords:** Carbon decomposition, Functional microbes, Nitrogen loading enrichment, Sea-level rise, Inundation, Oligohaline marsh

## Abstract

Increased nitrogen (N) loading and sea-level rise (SLR) are two dominant drivers of global change that threaten tidal marshes and the ecosystem services they provide, including the sequestration of organic carbon. Nevertheless, the mechanisms through which N loading enrichment, SLR inundation increase, and their combined effects impact the rates and pathways of soil organic carbon (SOC) mineralization in tidal marshes remain poorly understood. We utilized a factorial design in an oligohaline tidal marsh, utilizing *in situ* weirs to simulate SLR inundation increase by manipulating the duration of flooding with or without nitrogen enrichment as NaNO_3_ plus NH_4_Cl or with a combination of increased flood duration and nitrogen. After nearly 2 years, the addition of N increased total SOC mineralization (CMR), soil microbial Fe(III) reduction (FeRR), NO_3_^–^ reduction (NRR), and SO_4_^2–^ reduction (SRR) but decreased methanogenesis (MGR). The abiotic factor Fe(III)/Fe(II) ratio and dissolved organic carbon (DOC), and the biotic factors, β-glucosidase (BG), and phenol oxidase (PHO) activity explained the increased SOC mineralization rates following N enrichment. Increased flood duration did not change CMR, but increased flooding offset the stimulatory effects of N addition on CMR, FeRR, SRR, NRR and MGR. The contributions of Fe(III) reduction and SO_4_^2–^ reduction pathways to SOC mineralization increased in all experimental treatments, FeRR, SRR, NRR, and MGR were significantly positively correlated with the abundance of *Geobacter, dsr*A, *nrf*A, and *mcr*A. SLR inundation increase did not increase soil carbon loss in this oligohaline marsh and may counteract the simulation of soil C loss due to N enrichment.

## Highlights

• N addition enhances iron reduction, nitrate reduction and sulfate reduction.
• N addition increases C mineralization rates but decreases methanogenesis rates.
• Increased inundation decreases methanogenesis rates.
• Inundation counteracts promoting effect of N addition on C mineralization rates.

## Graphical abstract

**Figure:**
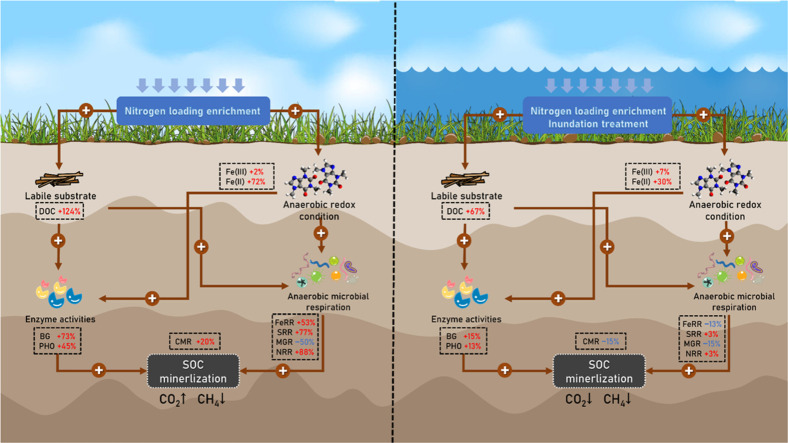

## 1 Introduction

Coastal tidal marshes are recognized for possessing high-value ecosystem services, including high primary productivity that results in the sequestration of significant quantities of organic carbon [1,2]. They are important sedimentary carbon sinks as a consequence of high unit area primary production, incomplete soil organic carbon (SOC) decomposition [3], and trapping and burial of vast amounts of C from autochthonous and allochthonous sources [2].

The ecosystem services of estuaries and tidal marshes in particular are increasingly challenged on a global scale by a variety of stressors. Human activity has substantially and directly increased the amount of reactive nitrogen (N) in the environment [4], and the impacts are particularly problematic in estuarine zones [5]. In addition, thermal expansion of the ocean and ice melt is expected to accelerate sea-level rise (SLR) significantly and, by extension, impact coastal zones over the coming century [6]. Hence, N eutrophication and SLR are dominant drivers of global change affecting coastal tidal wetland ecosystems. SOC stocks in coastal wetlands are controlled by a balance between C inputs from primary production and allochthonous deposition, and C losses including SOC mineralization (decomposition), and C export [7]. Wetland SOC mineralization is mediated by various heterotrophic microorganisms and pathways [8]. Electron acceptors such as nitrate (NO_3_^–^), ferric iron oxides (Fe(III)), sulfate (SO_4_^2–^), and CO_2_, and mineralization pathways including microbial Fe(III), SO_4_^2–^, and NO_3_^–^ reduction as well as methanogenesis are all potentially important and have a bearing on the ultimate end-products of anaerobic catabolism–CO_2_ and CH_4_ [9]. The distribution of these mineralized products is important because of the greater warming potential of CH_4_ and its explosive growth in the atmosphere [10]. Anthropogenic N loading can increase wetland SOC mineralization and reduce soil C storage [1,^5^,[11], [12], [13]. However, other studies have shown that N loading enhanced or had a neutral effect on soil carbon storage and/or belowground productivity [5,14]. Respiration rate was not directly limited by N in a peatland, but positive feedback on productivity and litter quality was observed, causing greater soil respiration in nutrient-rich sites [15]. But, uncertainty remains about the relationship between N addition, its form, and SOC mineralization. Experimental N additions have either increased or did not change SOC mineralization in coastal wetlands. SOC mineralization was unaffected by additions of urea in a coastal salt marsh [5] but significantly increased in response to fertilization of marsh sediment with NO_3_^–^ [1]. The effects of SLR on SOC mineralization in estuarine tidal marshes have received much attention. However, there are conflicting conclusions about the effects of increased flooding, possibly because of effects on primary productivity and/or salinity. Saltwater intrusion into tidal freshwater marshes from SLR has a great effect on SOC decomposition [16], [17], [18], but our study is focused on the singular effects of increased flooding, nitrogen enrichment, and their interaction.

The metabolic pathways are complex. The goal of our research was to study the impact of enhanced nitrogen loading and increased inundation caused by SLR on key components of the overall metabolic system. Specifically, in an estuarine oligohaline marsh, we investigated: (i) the effects of SLR inundation increase, N enrichment, and their combination on SOC mineralization rates and pathways, and (ii) assessed the relative importance of various mineralization pathways, and (iii) functional microbial abundance and enzyme activities.

## 2 Materials and methods

### 2.1 Study site

The present study was conducted at the Shanyutan (119°34′12″–119°40′40″ *E*, 26°00′36″–26°03′42″ *N*) which is the largest tidal wetland in the Min River Estuary of China. The native marsh species in the Shanyutan include *Cyperus malaccensis, Scirpus triqueter*, and *Phragmites australis*. The regional climate is warm and wet with mean annual temperature and precipitation of 19.6 °C and 1350 mm, respectively. The experimental site was an oligohaline *C. malaccensis* marsh stand averaging about 1.4 m in height and located at the central-western portion of the Shanyutan. Regular semi-diurnal tides flood the site to depths of 10–150 cm. The marsh is submerged for ∼5 h per 24-h cycle. Between May and December 2007, the average tidal water salinity was 4.2 ± 2.5 parts per thousand (ppt) and the soil total organic C at 0–50 cm depth was 19.7 ± 0.7 g kg^–1^ [19].

### 2.2 Experimental design and manipulation

**Fig. 1:**
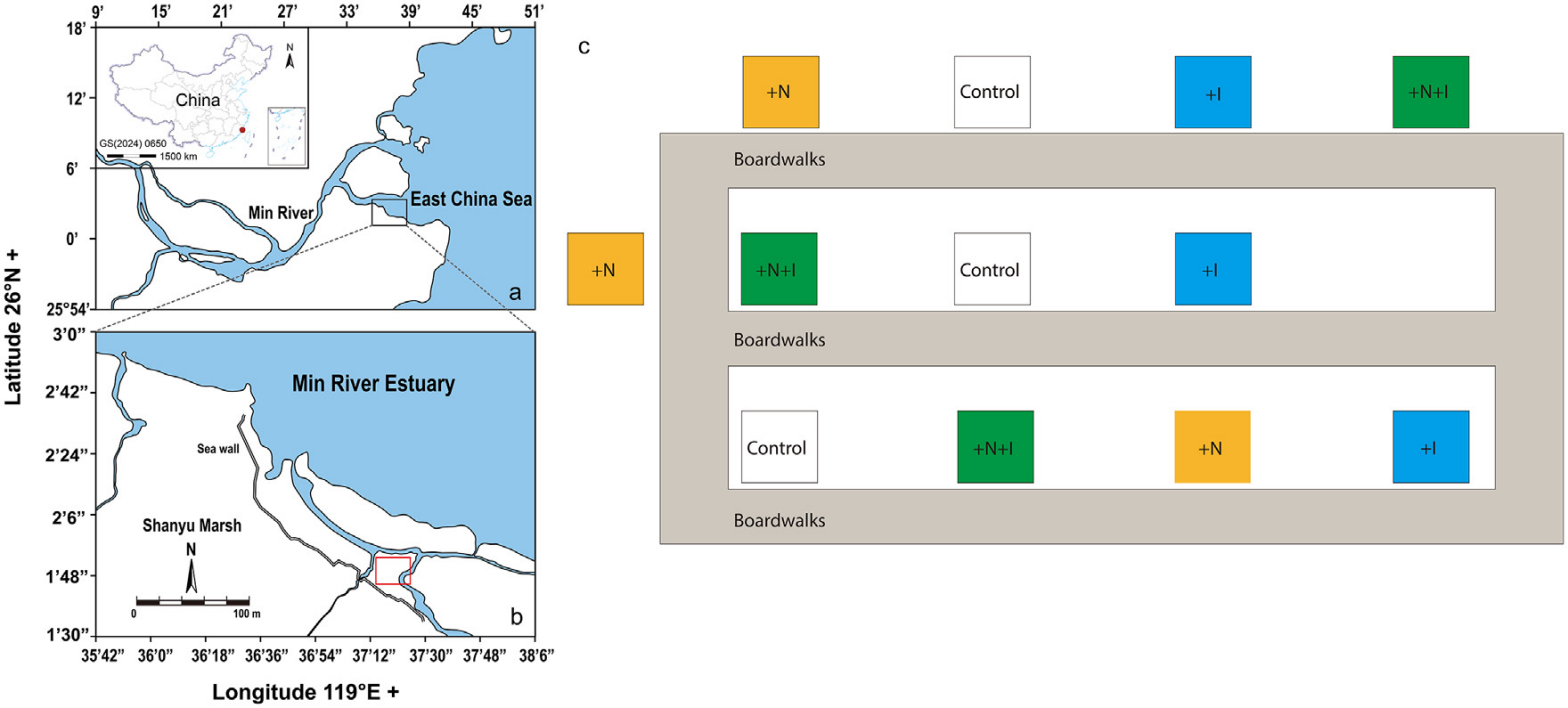
**Location of study area and experimental site (a, b) in oligohaline tidal marsh of Min River Estuary, Southeastern China.** +N: nitrogen addition treatments; +I: inundation increase treatments; +N+I: combined nitrogen addition plus inundation increase treatments.

There were four treatments with three replicates each, and they consisted of a control (Control), simulated SLR inundation increase (+I), nitrogen addition (+N), and combined N addition and inundation increase (+N+I). We used *in situ* weirs to simulate increased SLR. They were designed to increase flood depth and duration. This method has not previously been used to study the effects of increased flooding on SOC mineralization (decomposition) in coastal tidal marsh ecosystems. Here, the original design was modified by adding a floating ball valve to enhance the flexibility of control over flooding level and time (Fig. S1) [20]. In this manner, the inundation time was artificially increased to simulate a relatively higher mean sea level (MSL). Four treatments were randomly assigned to 12, 1 m^2^ experimental weirs separated 3 m apart to minimize cross-influence. A boardwalk was installed to minimize disturbance to the marsh surface (Fig. 1). Each weir consisted of four polypropylene walls 100 cm in height. The walls were inserted 35 cm into the soil. The weirs for the +I and +N+I treatments are depicted in Fig. S1 [20]. For each of these treatments, two holes were drilled in the sides facing the sea near ground level to allow tidal water to enter passively into the weir. In one hole a 5-cm-diameter one-way water flow control valve was installed. In the second hole, a 30 cm-long PVC pipe mounted with a floating ball valve was installed to control drainage during ebb tides. Control and +N weirs had only one hole without a check valve to allow unimpeded water flow. HOBO water level loggers (Onset Co., Cape Cod, MA, USA) were installed in the Control and +I (SLR) treatments. For the SLR treatment, the water level decreased slowly that of the Control, the inundation times were ∼3 × and ∼1.5 × longer than those for the Control during 1 wk in winter and 1 wk in summer 2019, respectively (Fig. S2). These inundation times are considered to be more representative of the flooding impacts that might be experienced due to future sea level rise [21].

Manipulations of MSL and nitrogen additions were initiated in November 2018 when the equivalent of 48 g m^–2^ yr^–1^ N was sprayed biweekly into the center of the +N weirs in the form of a mixed NaNO_3_ and NH_4_Cl solution in a 2:1 molar ratio based on nitrogen content, respectively, at the same ratio of wet NO_3_: NH_4_-N deposition around the study site (unpublished data). The cumulative N application rate was ∼10 × greater than the background N deposition in the study area [22] and ∼2 × higher than the total amount of N imported to the Minjiang River Estuary in the forms of N deposition plus N directly imported by the river [23]. For the +N and +N+I treatments, NaNO_3_ and NH_4_Cl were dissolved in 2 L tidal creek water and slowly sprayed over the weirs after the neap tide ebb to maximize retention. Then another 2 L tidal creek water was sprayed over the weirs to wash any N solution remaining on leaves onto the ground surface. Control weirs were watered with 4 L plain tidal creek water.

### 2.3 Soil and porewater sampling

Soil samples were collected on 3 dates: July 2019 (summer; after 240 d treatment), January 2020 (winter; after 420 d treatment), and August 2020 (summer; after 420 d treatment). On each date, three soil cores were collected at a depth of 10 cm. This depth was chosen because the most significant impact of inundation was anticipated to be concentrated within the upper sediment layers [24]. The soil cores were collected from each weir with a steel sediment sampler (Inner diameter = 5 cm) and returned to the laboratory within 6 h and stored at 4 °C in darkness. Additionally, subsamples of sediment for molecular genetic analyses were promptly snap-frozen in liquid nitrogen and stored at –80 °C. Within 1wk the cores from each weir were composited before further processing. In the laboratory, a portion of each soil sample was air-dried and pulverized in a ball mill.

Porewater sample was collected from each weir with a Rhizon sampler [25] at 10 cm below the surface. Each water sample was transferred to a polyethylene bottle and 0.2 mL saturated HgCl_2_ solution was added in the field as a preservative. The porewater samples were returned to the laboratory within 6 h, filtered through a 0.22-µm cellulose membrane filter (MilliporeSigma, Burlington, MA, USA), and stored at 4 °C before analyzing them within 1 wk by standard methods.

### 2.4 Soil and porewater geochemical analyses

The SOC content was measured with an elemental analyzer (Vario MAX CN; Elementar Analysensysteme GmbH, Langenselbold, Hesse, Germany) with 97%–105% recovery. Inorganic C as removed with 10% deoxygenated hydrochloride (HCl) before SOC determination. The total nitrogen (TN) content was also measured with an elemental analyzer (Vario MAX CN; Elementar Analysensysteme GmbH). Soil amorphous Fe(II) was extracted with 0.5 M deoxygenated HCl. Total amorphous Fe was extracted with a mixture of 0.5 M deoxygenated HCl plus 0.1 M hydroxylamine. The amorphous Fe(III) content was calculated as the difference between that of amorphous Fe and that of amorphous Fe(II). All Fe species were detected by the 1,10-phenanthroline photometric method [24]. The Fe(III)/Fe(II) ratios were calculated by dividing the Fe(III) content by the Fe(II) content. The total reduced sulfur (S) content was quantified by the methylene blue method after extraction with an extraction-diffusion apparatus [24].

Porewater pH and electrical conductivity (EC; mS cm^–1^) were measured *in situ* with a pH meter (No. pH400; Spectrum Technologies Inc., Paxinos, PA, USA) and an electrical conductivity meter (No. 2265FS; Spectrum Technologies Inc.), respectively. Dissolved organic carbon (DOC) was measured with a total organic C (TOC) analyzer (TOC-V CPH; Shimadzu Corp., Kyoto, Japan). The concentration of SO_4_^2–^ in the porewater was measured by ion chromatography (Dionex 2100; Thermo Fisher Scientific, Waltham, MA, USA). The SO_4_^2–^ detection limit was 8%. The concentrations of NH_4_^+^ and NO_3_^–^ in the porewater were spectrophotometrically determined by flow injection analyzer (SAN++; Skalar Analytical B.V., Breda, The Netherlands). The NO_3_^–^ concentrations were determined by the copper-cadmium reduction method, while the NH_4_^+^ concentrations were measured by the sodium hypobromite oxidation method.

### 2.5 Anaerobic soil incubation

The rates of soil microbial Fe(III) reduction (FeRR), NO_3_^–^ reduction (NRR), SO_4_^2–^ reduction (SRR), methanogenesis (MGR), and total SOC mineralization (CMR) were determined by anaerobic slurry incubation [17,24]. CMR and MGR were estimated from the ratios of [CO_2_ + CH_4_] and CH_4_ production to incubation time, respectively, with a Shimadzu GC-2010 gas chromatograph (GC; Shimadzu Corp.). FeRR, SRR, and NRR were estimated from the time series of Fe(II), TRS, and NH_4_^+^ production. For the MGR and CMR measurements, triplicate fresh ∼20-g soil samples were slurried with deoxygenated *in situ* surface water (2:1, w/w) under a nitrogen gas atmosphere, purged with N_2_ gas for 5 min, crimp-sealed, and incubated in 100-mL glass volumetric incubation bottles in the dark at ambient temperature (25 °C). Headspace gasses during 3-day incubations were analyzed for concentrations of CO_2_ and CH_4_. For the FeRR and SRR determinations, triplicate vials were sacrificed to determine Fe(II), TRS, and NH_4_^+^ accumulation over 3 days. The Fe(III) reduction rates were determined by the by the production of Fe(II) [26]. The rates of microbial sulfate reduction were determined by the production of TRS [27]. We cannot rule out the possibility that rapid reoxidation of TRS to SO_4_^2−^ was taking place in our slurries, leading to an underestimate of the role of sulfate reduction. However, given the anaerobic environment of the slurries, we assume that the reoxidation of TRS to SO_4_^2−^ was likely minimal in this experiment [28]. Nitrate reduction rates were estimated based on the accumulation of ammonium, as dissimilatory nitrate reduction to ammonium (DNRA) generally been observed to be dominant of nitrate reduction in coastal sediments [29,30]. CMR, FeRR, SRR, MGR, and NRR were calculated from linear regressions against time (*r*^2^ >0.80).

Each microbial respiration pathway rate was standardized to C units using the following theoretical stoichiometries Fe:C = 4:1, S:C = 1:2, CH_4_-C:C = 1:2, and N:C = 1:2 [23], and their contributions to SOC mineralization were then calculated.

### 2.6 Measurement of functional microbial abundance and enzymatic activity

Soil DNA was extracted from 0.25 g fresh soil with a PowerSoil DNA Extraction Kit (MO BIO Laboratories Inc., Carlsbad, CA, USA) according to the manufacturer's instructions. Extracted DNA quality and concentration were determined on 1% agarose gel and by NanoDrop spectrophotometry (Thermo Fisher Scientific). The bacterial 16S rRNA gene was targeted with 338F and 806R primers [32]. GM3 and 825R primers were used to quantify the abundance of the putative iron-reducing bacterium *Geobacter* [33]. The methyl-coenzyme M reductase alpha-subunit (*mcr*A) gene, which is associated with the reverse methanogenic pathway, was amplified using the primer pair *mcr*A-F/*mcr*A-R [34]. The quantification of the dissimilatory sulfite reductase subunit A (*dsr*A) gene, associated with the dissimilatory sulfate reduction pathway, was performed using the *dsr*A 290F and *dsr*A 660R primers [35]. Additionally, the nitrite reductase (*nrf*A) gene, linked to the nitrate reduction pathway, was amplified using the *nrf*A-2F and *nrf*A-2R primers in the extracted DNA [36]. All amplifications were conducted in 20-µL reaction mixtures containing 1 µL DNA template and 0.2 µM of each primer. The qPCR was performed in a Real-Time PCR System (Applied Biosystems 7500; Thermo Fisher Scientific).

The β−1,4-glucosidas (BG) activities was detected by fluorometry using 4-methylumbelliferone as the substrate. Phenol oxidase (PHO) activity was determined by colorimetry using *L*-3,4-dihydroxyphenylalanine as the substrate [37]. Fluorescence and absorbance were measured using a microplate reader (Synergy H4 Multi-Mode; BioTek Instruments, Winooski, VT, USA) in a 96-well black (for BG) or clear(for PHO) OptiPlate microplate (PerkinElmer, MA, USA).

### 2.7 Statistical analyses

All data were processed in *R* v. 4.1.2 (http://www.rproject.org). Differences between treatment means were considered statistically significant at *p* < 0.05. All datasets were assessed for normality using the Shapiro-Wilk test and were natural log-transformed before statistical analysis if necessary. The effects of N addition and increased inundation on CMR, FeRR, SRR, NRR, MGR, BG, and PHO activity, and the soil and porewater properties were tested by two-way ANOVA using the ‘*aov*’ function in *R*. Multiple comparisons across four treatments within a single period and multiple comparisons across three sampling dates within each treatment were evaluated by Tukey's HSD post hoc test. A Random Forest (RF) analysis was conducted in the ‘*linkET*’ package in *R* to identify statistically significant predictors of CMR, FeRR, SRR, MGR, and NRR. The % increases in mean squared error (MSE) were calculated to establish the relative importance of each biotic and abiotic variable in predicting CMR, FeRR, SRR, MGR, and NRR. Pearson's correlations among CMR, FeRR, SRR, MGR, and NRR and the biotic and abiotic variables were analyzed using the ‘*corrplot*’ package in *R*. We utilized structural equation modeling (SEM) to assess the relationships between N addition, inundation increases, soil and microbial properties, SOC mineralization pathway, and CMR. The SEM analyses were carried out using the “*lavaan*” package in *R*. Linear regressions were plotted to assess the relationships between the microbial respiration pathway rates and functional microbial abundance.

## 3 Results

### 3.1 Geochemical properties of soil and porewater

The treatments (+N, +I, and +N+I) had no effects on porewater electrical conductivity (EC), pH, Fe(III), or SO_4_^2–^, but these variables did vary by sampling date or season (Table 1). Variables insensitive to treatment and measured during winter (collected day 420) tended to be higher in EC, pH, or concentration than measurements made during summer (days 240 and 630) (Table 1). For example, SO_4_^2–^ increased from 0.3 to 1.5 between days 240 and 420, and dropped to 1.2 on day 630.

**Table 1:** **Effects of N addition and inundation increase on soil and porewater physicochemical properties [electrical conductivity, pH, SOC, TN, Fe(III), Fe(II), Fe(III)/Fe(II), TRS, DOC, SO**_**4**_^**2–**^**, NO**_**3**_^**–**^**, and NH**_**4**_^**+**^**] in oligohaline tidal marsh of the Min River Estuary, Southeastern China.**

Properties	Treatment days	Treatments				Two-way ANOVA analysis		
		Control	+N	+I	+N+I		F	*p*
Electrical conductivity (dS cm^−1^)	240	6.03 ± 0.70 b	6.24 ± 0.48 b	5.40 ± 0.79 b	5.72 ± 1.23 b	Treatment	2.581	0.077
	420	7.50 ± 0.65 a	6.74 ± 0.83 a	6.61 ± 0.18 a	8.11 ± 0.56 a	Time	34.100	< 0.001
	630	5.37 ± 0.53 c	4.65 ± 0.78 c	4.62 ± 0.44 c	5.09 ± 0.56 c	Interaction	1.102	0.390
pH	240	7.14 ± 0.21 a	6.87 ± 0.25 a	6.86 ± 0.50 a	6.92 ± 0.58 a	Treatment	0.158	0.923
	420	6.13 ± 0.12 b	6.33 ± 0.15 b	6.37 ± 0.18 b	6.39 ± 0.19 b	Time	48.126	< 0.001
	630	5.69 ± 0.23 c	5.48 ± 0.02 c	5.57 ± 0.03 c	5.64 ± 0.72 c	Interaction	0.440	0.845
SOC (mg g^−1^)	240	16.63 ± 1.40 Bb	22.54 ± 0.31 Ab	17.71 ± 0.36 Bc	20.92 ± 0.17 Bb	Treatment	11.459	< 0.001
	420	22.09 ± 4.13 Ba	23.98 ± 0.92 Ab	23.10 ± 1.82 Bb	23.37 ± 1.04 Bb	Time	51.422	< 0.001
	630	24.35 ± 1.87 Ba	37.20 ± 2.93 Aa	28.28 ± 5.80 Ba	30.24 ± 3.20 Ba	Interaction	2.569	0.046
TN (mg g^−1^)	240	1.71 ± 0.12 Cb	2.26 ± 0.03 Ab	1.85 ± 0.07 BCb	2.13 ± 0.13 ABb	Treatment	13.816	< 0.001
	420	2.15 ± 0.29 Cb	2.29 ± 0.11 Ab	2.24 ± 0.14 BCb	2.27 ± 0.10 ABb	Time	56.129	< 0.001
	630	2.35 ± 0.12 Ca	3.09 ± 0.08 Aa	2.57 ± 0.31 BCa	2.80 ± 0.23 ABb	Interaction	1.970	0.110
Fe(III) (µmol g^−1^)	240	151.16 ± 14.15 a	142.89 ± 7.07 a	175.53 ± 45.51 a	176.05 ± 26.85 a	Treatment	0.678	0.574
	420	151.94 ± 17.31 b	168.76 ± 11.47 b	179.66 ± 46.07 b	180.89 ± 16.67 b	Time	10.509	0.001
	630	131.03 ± 24.07 b	132.10 ± 17.38 b	127.48 ± 27.36 b	106.17 ± 26.37 b	Interaction	1.011	0.441
Fe(II) (µmol g^−1^)	240	7.03 ± 1.00 B	14.63 ± 3.60 A	8.13 ± 0.70 B	9.10 ± 0.43 B	Treatment	10.608	< 0.001
	420	6.28 ± 1.33 B	10.01 ± 2.24 A	6.55 ± 0.40 B	7.92 ± 1.65 B	Time	3.360	0.052
	630	6.98 ± 1.08 B	10.26 ± 3.12 A	8.81 ± 1.52 B	9.37 ± 2.63 B	Interaction	1.271	0.307
Fe(III)/Fe(II)	240	21.86 ± 4.29 Ab	10.25 ± 3.02 Bb	21.56 ± 5.26 Ab	19.36 ± 2.85 Bb	Treatment	5.766	0.004
	420	24.78 ± 4.67 Aa	17.49 ± 4.21 Ba	27.67 ± 8.23 Aa	23.25 ± 3.11 Ba	Time	9.629	0.001
	630	19.41 ± 6.35 Ab	13.34 ± 2.60 Bb	14.65 ± 3.49 Ab	12.11 ± 5.38 Bb	Interaction	1.030	0.431
TRS (µmol g^−1^)	240	0.59 ± 0.15 Bb	0.38 ± 0.10 Bc	0.47 ± 0.18 Bb	0.65 ± 0.18 Ac	Treatment	5.686	0.004
	420	1.29 ± 0.56 Ba	1.47 ± 0.14 Ba	0.75 ± 0.22 Bb	2.53 ± 1.20 Aa	Time	15.551	< 0.001
	630	0.66 ± 0.17 Bb	0.83 ± 0.11 Bb	1.10 ± 0.27 Ba	1.38 ± 0.46 Ab	Interaction	2.516	0.049
DOC (mmol L^−1^)	240	24.73 ± 3.69 Ca	57.22 ± 4.13 Aa	41.10 ± 3.04 Ba	41.12 ± 6.02 Ba	Treatment	59.271	< 0.001
	420	20.57 ± 4.68 Ca	28.24 ± 5.22 Ab	23.01 ± 1.62 Bb	19.07 ± 1.45 Bb	Time	69.328	< 0.001
	630	14.48 ± 2.96 Cb	48.30 ± 3.21 Aa	44.72 ± 3.14 Ba	39.77 ± 5.68 Ba	Interaction	10.466	< 0.001
SO_4_^2–^ (mmol L^–1^)	240	0.19 ± 0.00 b	0.49 ± 0.22 b	0.15 ± 0.06 b	0.36 ± 0.17 b	Treatment	2.787	0.062
	420	1.67 ± 0.51 a	1.64 ± 0.87 a	1.19 ± 0.09 a	1.36 ± 0.06 a	Time	46.365	< 0.001
	630	1.29 ± 0.15 a	1.42 ± 0.14 a	0.97 ± 0.06 a	1.22 ± 0.13 a	Interaction	0.364	0.895
NO_3_^–^ (µmol L^–1^)	240	2.26 ± 1.01 B	4.67 ± 0.99 A	1.94 ± 1.28 BC	3.08 ± 0.27 AB	Treatment	14.721	< 0.001
	420	1.61 ± 0.28 B	4.82 ± 1.77 A	1.45 ± 0.01 BC	3.70 ± 1.81 AB	Time	0.149	0.862
	630	2.44 ± 0.53 B	5.03 ± 0.52 A	1.87 ± 0.79 BC	3.20 ± 1.70 AB	Interaction	0.286	0.938
NH_4_^+^ (µmol L^–1^)	240	40.00 ± 4.41 Ba	53.33 ± 8.33 Aa	33.89 ± 5.09 Ba	51.67 ± 8.33 Aa	Treatment	9.489	< 0.001
	420	16.67 ± 6.01 Bb	23.89 ± 6.74 Ab	13.89 ± 0.96 Bb	23.89 ± 5.85 Ab	Time	58.499	< 0.001
	630	16.94 ± 6.14 Bb	23.89 ± 3.47 Ab	21.67 ± 5.77 Bb	31.11 ± 8.22 Aa	Interaction	1.160	0.360

Different capital letters indicate significant differences across four treatments (two-way ANOVA; *p* < 0.05). Different lowercase letters indicate significant differences across three sampling dates within each treatment (one-way ANOVA; *p* < 0.05).

+N: nitrogen addition treatment; +I: inundation increase treatment; +N+I: combined nitrogen addition plus inundation increase treatment.

The +N treatment had a significant effect on all other geochemical properties and, except for the Fe(III)/Fe(II) ratio and TRS, produced a significantly greater response than other treatments and controls. Geochemical response variables were more similar between controls and the +I treatment than they were to either +N or +N+I treatments. For example, the mean NO_3_^–^ of control and +I treatments were 2.1 and 1.7 µmol L^–1^ compared to means of 4.9 and 3.4 umol L^–1^ in the +N and +N+I treatments, respectively (Table 1). The mean NH_4_^+^ concentrations (16.7 ± 6 to 53.3 ± 8 µmo L^–1^, depending on treatment and time) were about an order greater than the mean NO_3_^–^ concentrations (1.7–4.9 µmol L^–1^) (Table 1).

DOC had the greatest response to the +N treatment, increasing 37% to 233% above the level of the controls, depending on time, followed by NO_3_^–^, which increased by 139% above controls. TRS initially had a negative response to +N, decreasing 53% below the controls by day 240, but it eventually increased to 26% above controls by day 630 (Table 1). The Fe(III)/Fe(II) ratio had a consistently negative response to +N, decreasing initially by 53% and eventually by 31% below control levels by day 630.

Among geochemical parameters that were correlated, the strongest (Pearson Correlation Coefficient, n = 12) was between SOC and DOC (*r* = 0.45, *p* < 0.05). The correlation between NH_4_^+^ and TRS was negative (*r* = −0.41, *p* < 0.05) and negative between NH_4_^+^ and SOC (*r* = −0.33, *p* < 0.05).

### 3.2 Rates and contributions of different SOC mineralization pathways

**Fig. 2:**
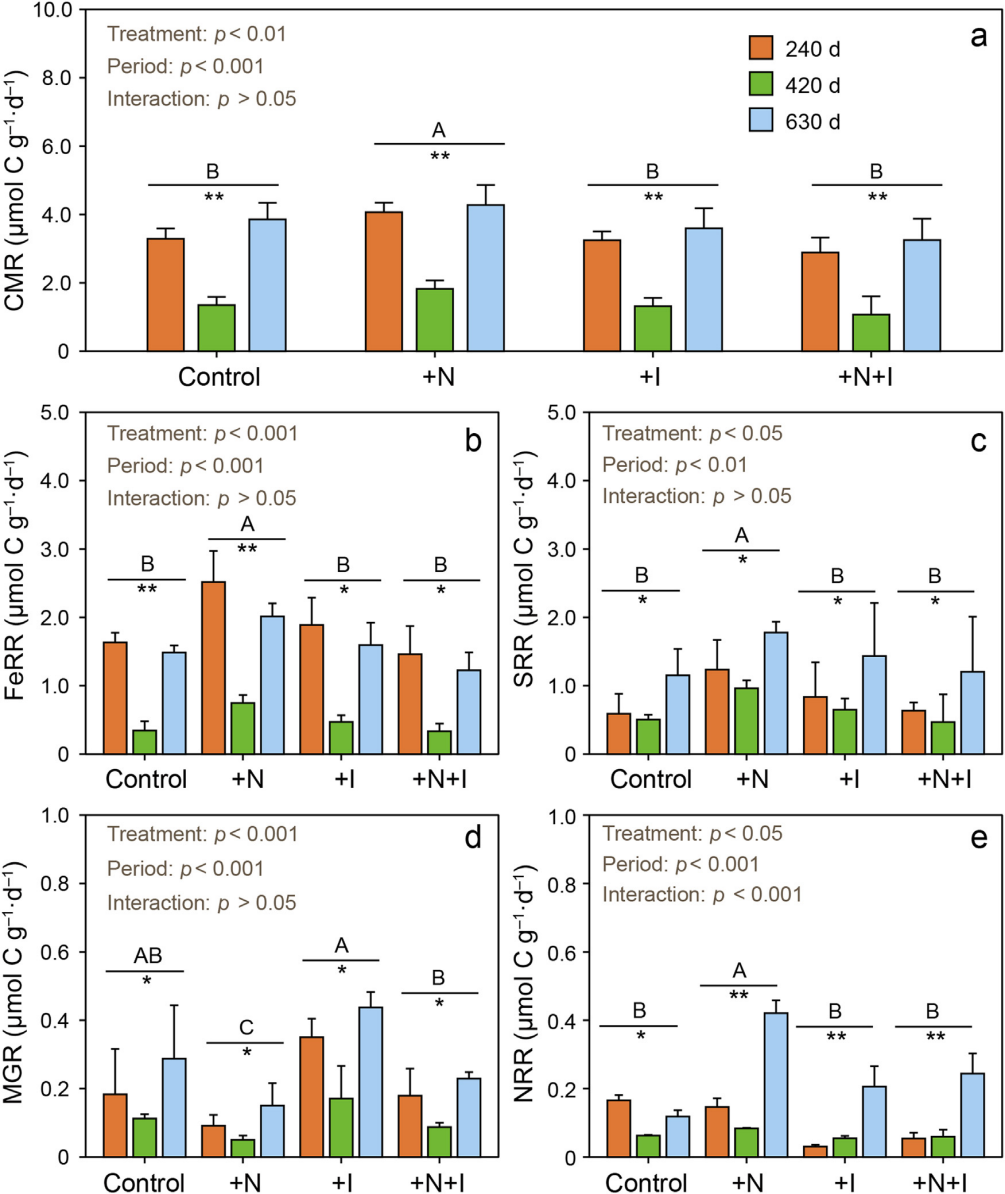
**Effects of N addition and inundation increase on CMRs, FeRRs, SRRs, MGRs, and NRRs at 240 d, 420 d, and 630 d (n = 3).** Different capital letters indicate significant differences across treatments (*p* < 0.05; Tukey's post hoc test). Asterisks denote significant differences across three sampling dates within each treatment (*p* < 0.05; Tukey's post hoc test). +N: nitrogen addition treatments; +I: inundation increase treatments; +N+I: combined nitrogen addition plus inundation increase treatments. CMR: carbon mineralization rates; FeRR: Fe(III) reduction rates; SRR: sulfate reduction rates; MGR: methanogenesis rates; NRR: nitrate reduction rates.

The +N treatments significantly increased the CMR while the +I and +N+I treatments did not (Fig. 2a). The +N treatments significantly increased the FeRR, SRR, and NRR (Fig. 2b–e) but significantly decreased the MGR (Fig. 2d). The +I and +N+I treatments did not significantly change the MGR (Fig. 2d), FeRR, SRR, or NRR (Fig. 2b–e). The CMR and the rates of the four pathways of SOC mineralization in the control and three treatments were significantly lower in the winter (at 420 d) than in the summer (at 240 d and 630 d) (Fig. 2a–d).

**Fig. 3:**
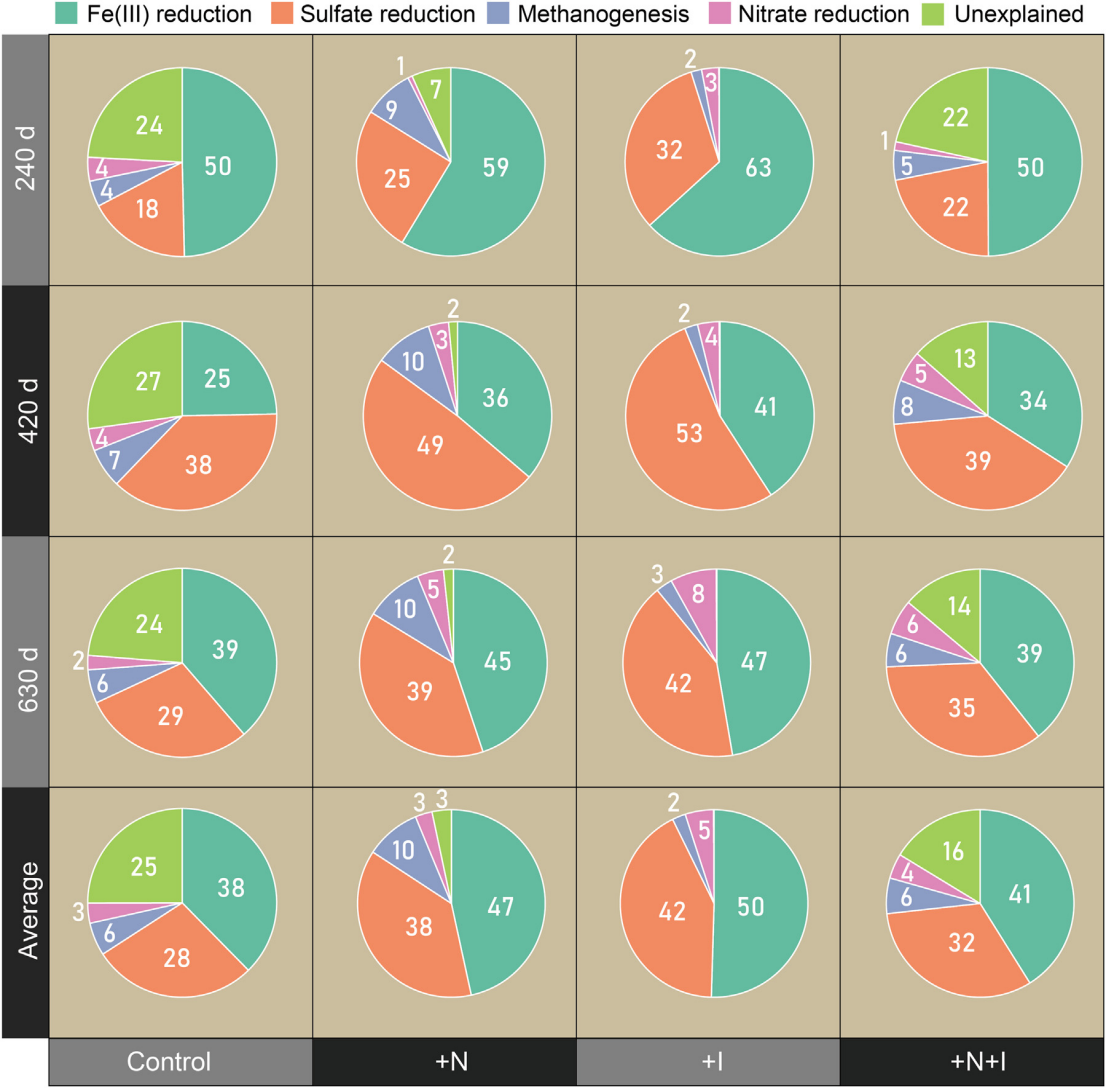
**Contributions (%) of FeRRs, SRRs, MGRs, NRRs and unexplained microbial respiration pathways to CMRs.** CMR: carbon mineralization rates; FeRR: Fe(III) reduction rates; SRR: sulfate reduction rates; MGR: methanogenesis rates; NRR: nitrate reduction rates.

In the control treatment, the Fe(III) reduction pathway dominated soil SOC mineralization at 240 d and 630 d whereas the SO_4_^2–^ reduction pathway dominated SOC mineralization at 420 d (Fig. 3). Under the +N, +I, and +N+I treatments, the Fe(III) reduction contributions increased by 12%, 9%, and 3%, respectively, while the SO_4_^2–^ reduction contributions increased by 14%, 10%, and 4%, respectively, compared with the control. Under the +N, +N+I, and +I treatments, the methanogenesis contribution increased by 4%, decreased by 4%, and did not change, respectively, relative to the control (Fig. 3).

### 3.3 Microbial activity and abundance

**Fig. 4:**
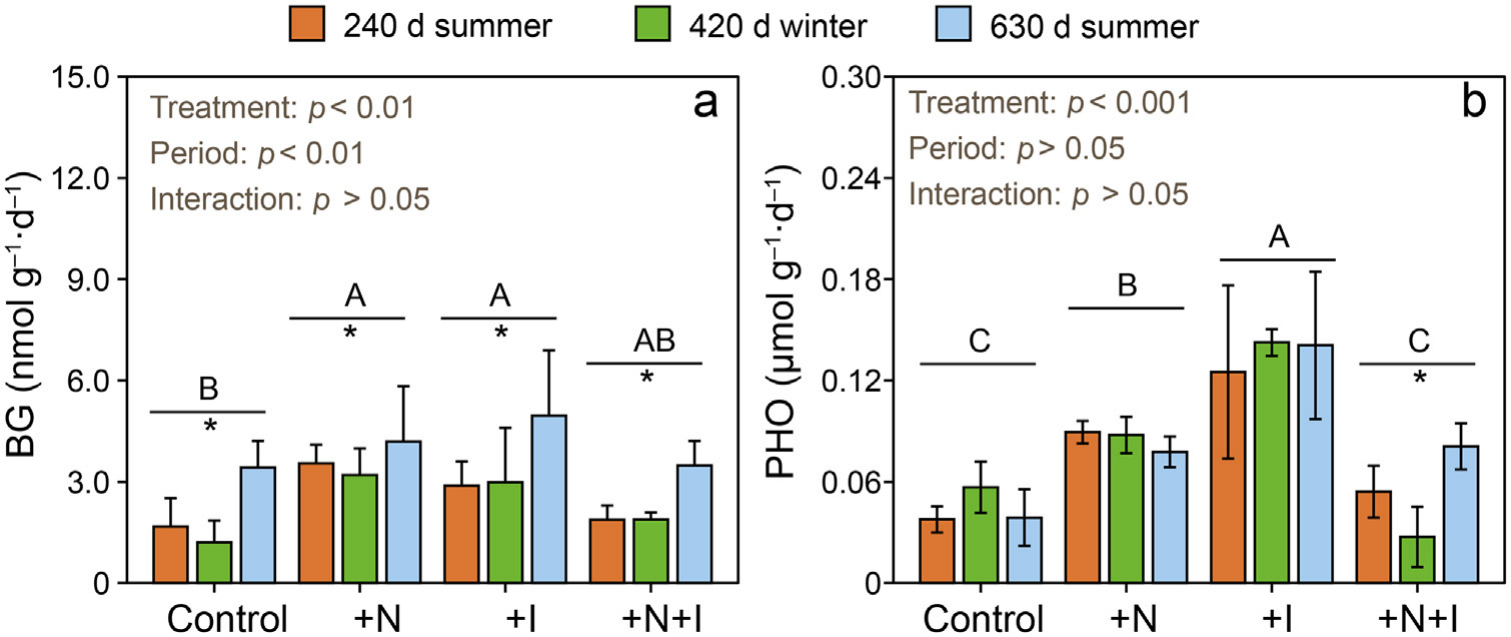
**Effects of N addition and inundation increase on BG (β−1,4-glucosidase) and PHO (phenol oxidase) activity at 240 d, 420 d, and 630 d (n = 3).** Different capital letters indicate significant differences across treatments (*p* < 0.05; Tukey's post hoc test). Asterisks denote significant differences across three sampling dates within each treatment (*p* < 0.05; Tukey's post hoc test). +N: nitrogen addition treatments; +I: inundation increase treatments; +N+I: combined nitrogen addition plus inundation increase treatments.

The +N and +I treatments significantly increased β−1,4-glucosidase (BG) and phenol oxidase (PHO) activity whereas the +N+I treatments did not (Fig. 4a and b). The +N treatments significantly increased the abundance of *Geobacter, dsr*A, and *nrf*A but did not change the abundance of *mcr*A (Table 2). The +I treatment significantly increased the abundance of *Geobacter* and *dsr*A but did not change the abundance of bacteria, *mcr*A, or *nrf*A. The +N+I treatments did not significantly affect the abundance of *Geobacter, dsr*A, *mcr*A, or *nrf*A. For all treatments including the control, the abundance of *Geobacter* was significantly higher in winter (at day 420) than in summer (at days 240 and 630). For all treatments including the control, the abundance of *mcr*A was significantly lower in winter (at day 420) than in summer (at days 240 and 630) (Table 2).

**Table 2:** **Effects of N addition and inundation increase on abundances of*****Geobacter, dsr*****A,*****mcr*****A, and*****nrf*****A in soils of oligohaline tidal marsh of the Min River Estuary, Southeastern China.**

Properties	Treatment days	Treatments				Two-way ANOVA analysis		
		Control	+N	+I	+N+I		F	*p*
Bacteria (10^9^ copies g^–1^)	240	5.10 ± 0.68 Bb	6.70 ± 1.26 Ac	5.28 ± 1.15 Bb	6.36 ± 1.01 Bb	Treatment	13.653	< 0.001
	420	32.19 ± 3.63 Ba	45.70 ± 7.41 Aa	28.78 ± 2.53 Ba	22.16 ± 0.81 Ba	Time	107.129	< 0.001
	630	4.18 ± 2.40 Bb	22.24 ± 8.49 Ab	7.37 ± 1.17 Bb	7.24 ± 3.93 Ba	Interaction	3.778	0.014
*Geobacter* (10^5^ copies g^–1^)	240	4.42 ± 1.79 Cb	54.09 ± 7.36 Aa	36.52 ± 9.03 Ba	10.82 ± 2.02 Ca	Treatment	69.079	< 0.001
	420	8.38 ± 3.00 Ca	21.75 ± 9.83 Ab	14.04 ± 1.83 Bb	6.00 ± 0.71 Cb	Time	36.512	< 0.001
	630	6.41 ± 1.11 Ca	76.32 ± 14.09 Aa	66.99 ± 9.78 Ba	8.50 ± 2.07 Ca	Interaction	12.309	< 0.001
*dsr*A (10^4^ copies g^–1^)	240	1.40 ± 0.78 Bb	3.06 ± 1.65 Ab	4.26 ± 2.00 Ac	1.52 ± 0.69 Bb	Treatment	16.711	< 0.001
	420	4.00 ± 0.95 Ba	12.18 ± 4.29 Aa	12.10 ± 2.71 Aa	4.31 ± 0.63 Ba	Time	20.169	< 0.001
	630	7.44 ± 4.77 Ba	15.75 ± 1.91 Aa	8.17 ± 1.63 Ab	5.51 ± 0.75 Ba	Interaction	3.525	0.019
*mcr*A (10^4^ copies g^–1^)	240	44.97 ± 2.08 a	48.65 ± 9.77 a	53.73 ± 5.49 a	48.50 ± 5.69 a	Treatment	1.430	0.269
	420	1.59 ± 0.65 b	0.70 ± 0.31 b	3.82 ± 0.37 b	1.07 ± 0.07 b	Time	256.410	< 0.001
	630	29.65 ± 2.85 a	24.24 ± 5.70 a	34.81 ± 8.08 a	29.66 ± 7.11 a	Interaction	0.870	0.537
*nrf*A (10^4^ copies g^–1^)	240	5.11 ± 0.70 Ba	4.12 ± 0.42 Ab	2.32 ± 0.39 Bb	2.10 ± 0.49 Bb	Treatment	12.678	< 0.001
	420	3.63 ± 0.75 Bb	7.64 ± 1.81 Ab	3.26 ± 1.03 Bb	4.21 ± 0.30 Bb	Time	31.185	< 0.001
	630	6.27 ± 0.75 Ba	11.17 ± 1.68 Aa	6.50 ± 1.16 Ba	8.55 ± 2.04 Ba	Interaction	2.919	0.038

Different capital letters indicate significant differences across four treatments (two-way ANOVA; *p* < 0.05). Different lowercase letters indicate significant differences across three sampling dates within each treatment (one-way ANOVA; *p* < 0.05).

+N: nitrogen addition treatment; +I: inundation increase treatment; +N+I: combined nitrogen addition plus inundation increase treatment.

### 3.4 Relationships among SOC mineralization, environmental factors, and functional microbial abundance

**Fig. 5:**
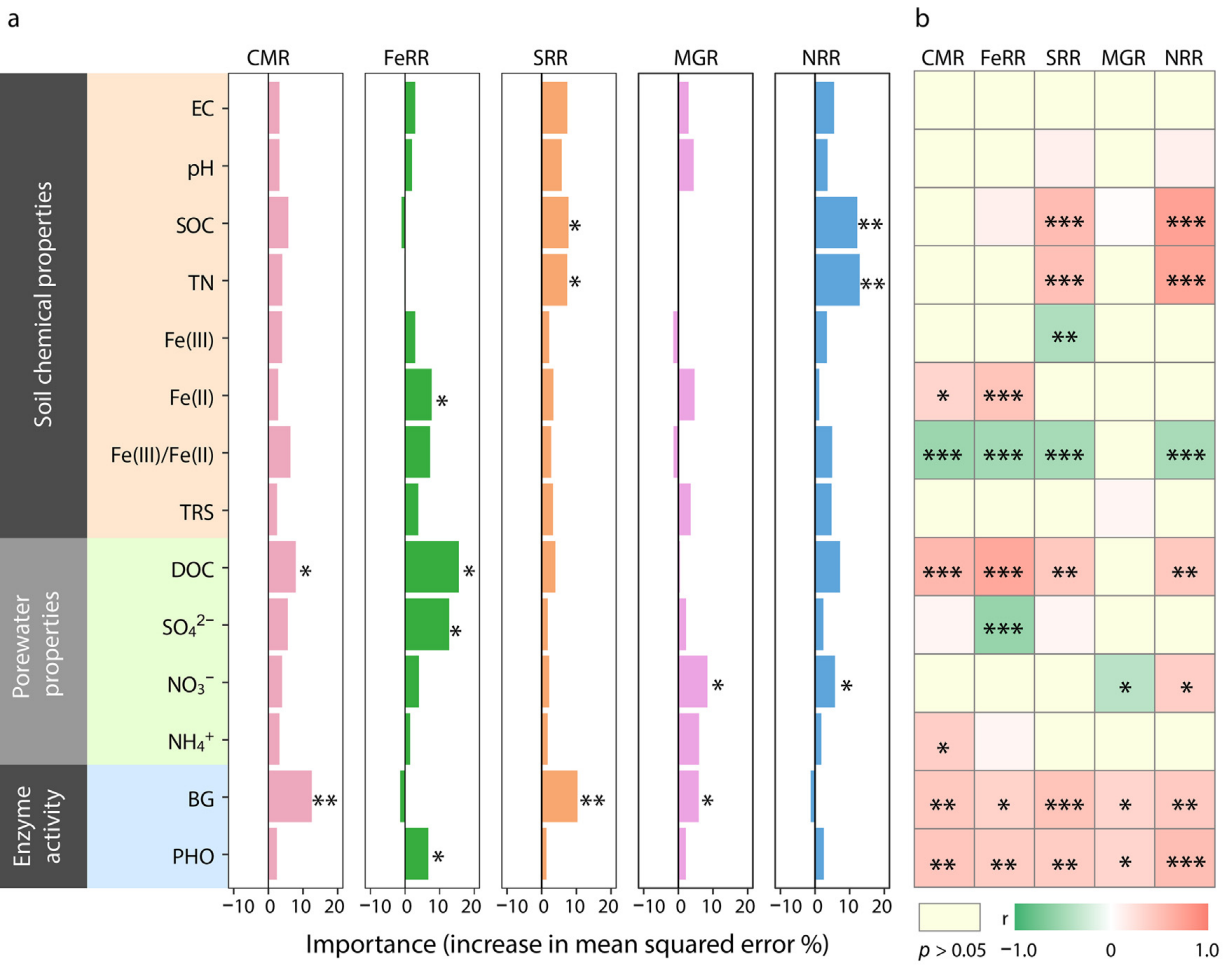
(a) % increases in mean squared error (MSE) (%) based on Random Forest (RF) analysis. (b) Relationships among CMR, FeRR, SRR, MGR, and NRR and selected biotic and abiotic factors. EC, electrical conductivity; SOC, soil organic carbon; TN, total nitrogen; TRS, total reduced sulfur; DOC, dissolved organic carbon; BG, β−1,4-glucosidase; CBH, cellobiohydrolase; CMR: carbon mineralization rates; FeRR: Fe(III) reduction rates; SRR: sulfate reduction rates; MGR: methanogenesis rates; NRR: nitrate reduction rates. *p < 0.05; **p < 0.01.

The Random Forest analysis suggested that the biotic and abiotic factors most strongly influencing CMR, FeRR, SRR, MGR, and NRR were BG and PHO activities, DOC, NO_3_^–^ and SO_4_^2–^ concentrations, Fe(II), SOC and TN contents (Fig. 5a). The CMR, FeRR, SRR, MGR, and NRR rates were positively correlated with BG and PHO activities (Fig. 5b). All rates except that of MGR were negatively correlated with the Fe(III)/Fe(II) ratio, and positively correlated with the DOC concentrations (Fig. 5b). FeRR was positively correlated with Fe(II) content but negatively correlated with SO_4_^2–^concentrations (Fig. 5b). SRR was positively correlated with TN and SOC contents, and DOC concentrations but negatively correlated with Fe(III) content (Fig. 5b). MGR was negatively correlated with NO_3_^–^ concentrations (Fig. 5b). NRR was positively correlated with SOC, TN content, DOC and NO_3_^–^ concentrations (Fig. 5b).

**Fig. 6:**
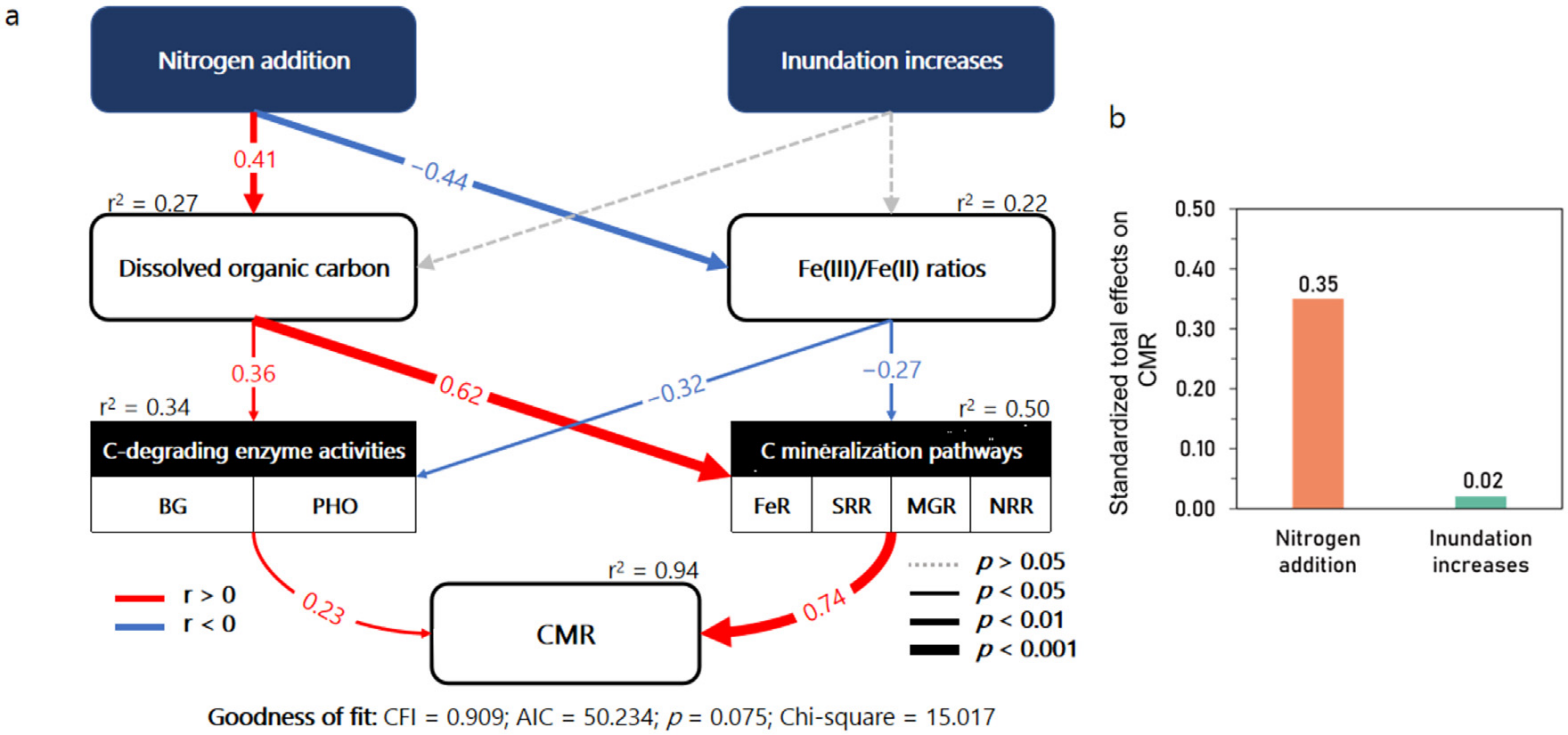
(a) Structural equation model (SEM) describing direct and indirect effects of N addition and inundation increases on soil CMRs. (b) Represent standardized indirect effects of N addition and inundation increase on CMRs, respectively. Values adjacent to column represent standardized coefficients in SEM. Values above rectangles indicate explained variance (*r*^2^) of variables. Numbers on arrows indicate standardized path coefficients. Arrow width indicates significance. CMR: C mineralization rates; FeRR: Fe(III) reduction rates; SRR: sulfate reduction rates; MGR: methanogenesis rates; NRR: nitrate reduction rates.

**Fig. 7:**
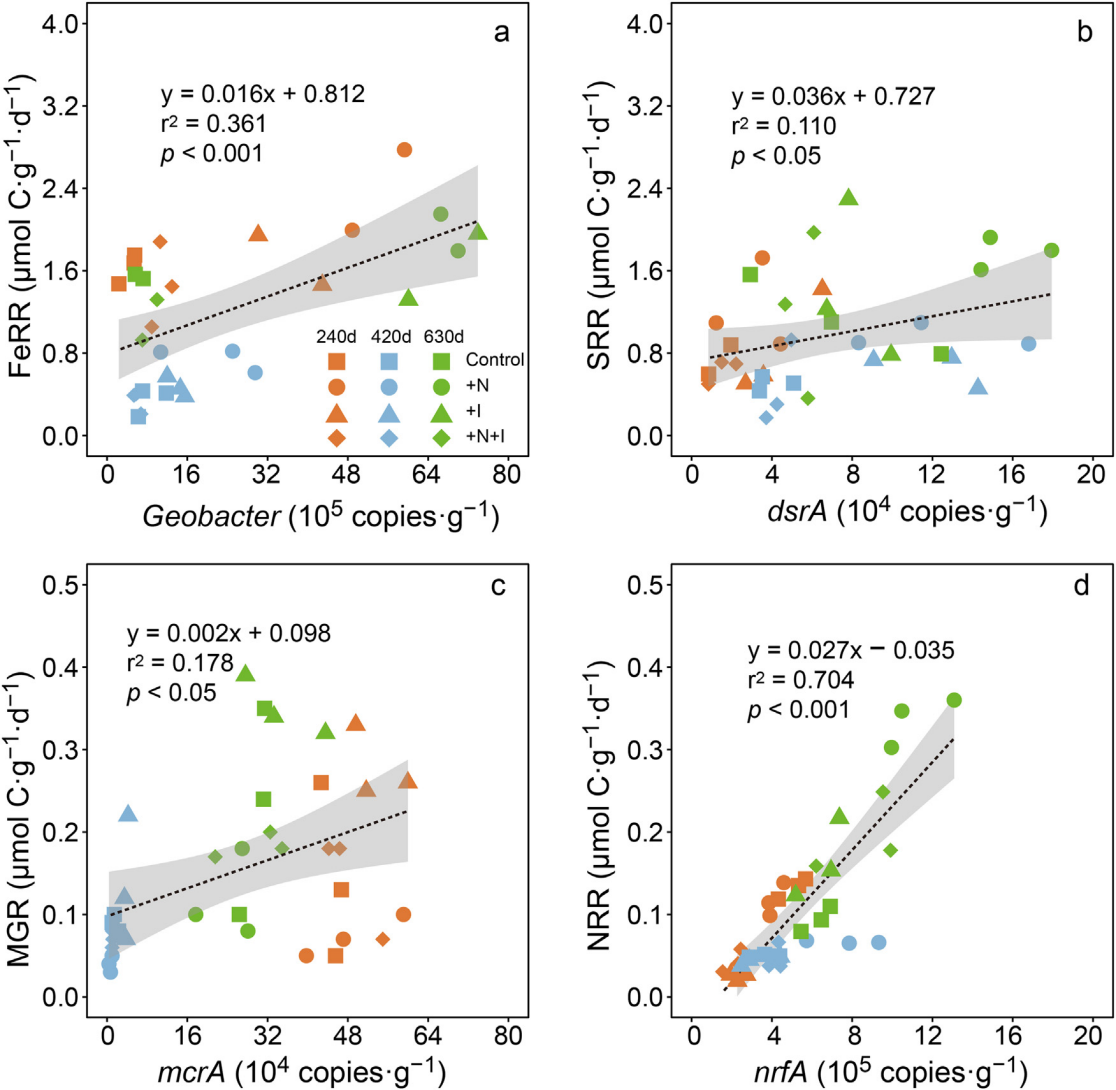
**Correlations among FeRRs, SRRs, MGRs, and NRRs and abundances of functional genes.** +N: nitrogen addition treatments; +I: inundation increase treatments; +N+I: combined nitrogen addition and inundation increase treatments. FeRR: Fe(III) reduction rates; SRR: sulfate reduction rates; MGR: methanogenesis rates; NRR: nitrate reduction rates.

The results of the SEM analysis suggested that N addition increased DOC but decreased the Fe(III)/Fe(II) ratio (Fig. 6a). The increases in DOC stimulated C-degrading enzyme (BG and PHO) activity and the rates of the SOC mineralization pathways whereas the decreases in the Fe(III)/Fe(II) ratio had the opposite effects. Both the C-degrading enzyme activity levels and the rates of the SOC mineralization pathways positively contributed to the SOC mineralization rates (CMR). Increased Inundation did not significantly influence DOC or the Fe(III)/Fe(II) ratio. The standardized total effects of N addition and increased inundation on the SOC mineralization rates were 0.35 and 0.02, respectively (Fig. 6b). There were significant positive correlations between biogeochemical rates and the abundances of their respective functional gene copies (Fig. 7). The Fe(III) reduction rates were significantly, and positively correlated with the abundance of *Geobacter*. The nitrate reduction rates were positively correlated with the *nrf*A abundance (Fig. 7d), and sulfate reduction rates and methanogenesis rates were marginally correlated (*p* < 0.05) with the abundances of *dsr*A and *mcr*A, respectively (Fig. 7b and c).

## 4 Discussion

The positive, negative, and neutral influences of increased saltwater flooding on SOC mineralization in tidal freshwater wetlands were previously reviewed [17]. By contrast, far less has been reported on the effects of increased flooding on SOC mineralization in tidal brackish and salt wetlands. In laboratory experiments, Lewis et al. [38] reported that the SOC mineralization was 18% lower in response to 20 h d^–1^ flooding than 4 h d^–1^ flooding of soils from *Avicennia germinans* mangrove forests and *Juncus roemerianus* salt marshes. Similarly, Chambers et al. [16] found in a mesocosm study that increasing tidal inundation decreased the soil CO_2_ production rate by 35%–37%. However, increasing only the salinity increased the soil CO_2_ production rate by an average of 17%–21%, while the combination of increased flooding and elevated salinity synergistically decreased the rate of CO_2_ production by 19%–26% below that of the control. The difficulty of interpreting these results underscores the need for additional research on the mechanisms by which SLR affects SOC mineralization.

The effects of SLR-saltwater intrusion on the rates and contributions of different SOC mineralization pathways have also received attention. Weston et al. [39] found that the contributions of SO_4_^2–^ reduction and methane production to SOC mineralization increased from 4% to 10% and decreased from 8% to 5%, respectively, along a saline (0–12 ppt) gradient in the Delaware River Estuary. Luo et al. [24] reported that the contributions of SO_4_^2–^ reduction to SOC mineralization increased, while the contributions of Fe(III) reduction and CH_4_ production to SOC mineralization decreased, along a low-level salinity gradient (0.1–3.3 ppt) in the MinJiang River Estuary, China. At present, however, the effects of SLR on the pathways of SOC mineralization have not been reported.

N enrichment and SLR often occur simultaneously in estuarine tidal wetlands, but the interactive effects on SOC mineralization and pathways are poorly understood. In a factorial study of the effects of combinations of nitrogen and flooding using *in situ* tidal marsh mesocosms, it was shown that N addition enhanced plant growth, particularly at sea levels where plants were most stressed by flooding [40]. In a saltmarsh mesocosm study, it was shown that increased inundation frequency led to more CH_4_ emissions and DOC loss but reduced CO_2_ emissions, but the effect of inundation was reduced by added nitrogen [41].

### 4.1 Effects of N addition on wetland SOC mineralization

In the present study, the addition of NaNO_3_ plus NH_4_Cl significantly increased the SOC mineralization rate as well as the Fe(III), SO_4_^2–^, and NO_3_^–^ reduction rates in an oligohaline tidal marsh. The effects of N enrichment on increases in the SOC mineralization rate (soil respiration or soil C emission) were addressed in previous studies on coastal wetlands [42] and inland wetlands [43]. However, there is uncertainty concerning the relationship between N addition and SOC mineralization (decomposition and CO_2_ production). Morris and Bradley [42] speculated that an increase in CO_2_ production may be a result of an increase in labile organic matter production and decay rather than an increase in stable C pool decay. A study by Vivanco et al. [5] in a coastal salt marsh found that potential SOC mineralization did not change with N addition, but in laboratory incubations, additions of NH_4_NO_3_ were shown to inhibit CO_2_ production from soil extracted from a freshwater marsh, which was attributed to C-limitation of N-induced microbial activity [12]. A long-term study of urea-N additions showed accelerated decomposition of light carbon fractions and stabilization of heavy fractions, but there were no statistically significant changes in total SOC decomposition [44].

The effects of N addition may vary with N-form (NO_3_^–^ *vs*. NH_4_^+^) and concentration. NH_4_Cl additions were shown to reduce CO_2_ emission from fen and especially low-N peat soil, which was attributed to soil acidification [45]. In laboratory incubations, NH_4_^+^ addition reduced CO_2_ efflux in acid soil, and NO_3_^–^ additions also lowered CO_2_ efflux but only when it was added a high level [46]. The dissolved inorganic carbon (DIC) production rate was significantly increased by adding NO_3_^–^ [1]. In laboratory experiments, both NO_3_^–^ and NH_4_^+^ additions to coastal wetland soil were found to enhance soil organic matter (SOM) decomposition, with NO_3_^–^ exhibiting a stronger stimulatory effect than NH_4_^+^ [11]. However, NO_3_^–^ had a stronger stimulatory effect to SOC mineralization compared to NH_4_^+^, since the NO_3_^–^ was also a terminal electron acceptor for SOC mineralization [11].

SOC mineralization is the result of multiple pathways, including Fe(III), SO_4_^2–^, and NO_3_^–^ reduction as well as methanogenesis. Here, the addition of a mixture of NaNO_3_ and NH_4_Cl increased the rates of Fe(III), SO_4_^2–^, and NO_3_^–^ reduction but not methanogenesis (Fig. 2). Hence, N loading increases SOC mineralization. The mechanisms by which N addition affects SOC mineralization involve enzyme activity [47], soil C storage [11], and pH [46] among others. In the present study, N addition significantly increased DOC (Fig. 6) probably as a consequence of increased root exudates [20]. Coastal wetlands appear to be universally limited by nitrogen [48] and a logical conclusion is that exudates from the root are proportional to primary production [49]. As a proof, N addition significantly increased belowground biomass [20].

N loading decreased the Fe(III)/Fe(II) ratio, indicating a decrease in soil redox potential [31]. This was linked to an increase in SOC mineralization rate and C-degrading enzyme activity (Fig. 6). As N addition lowered the Fe(III)/Fe(II) ratio, increased N loading facilitated the formation of anaerobic redox conditions, conducive to anaerobic microbial respiration (Fig. 6). Previous studies reported that the Fe(II) content was positively associated with C-degrading enzyme activity [50,51], and the present study likewise showed that an decrease in the Fe(III)/Fe(II) ratio influenced C-degrading enzyme activity. Fe(II) may enhance phenol oxidase activity by increasing hydroxyl radical production [50], and we found parallel patterns of change in β−1,4-glucosidase and phenol oxidase activity (*r*^2^ = 0.12; *p* < 0.05). Phenol oxidase degrades phenol compounds that are toxic to hydrolytic enzymes in the soil [52]. In summary, N enrichments increased C-degrading enzyme activity and, ultimately, SOC mineralization (Fig. 6).

In the present study, the addition of a mixture of NaNO_3_ and NH_4_Cl significantly decreased the rate of methanogenesis (Fig. 2). Results of previous work on nitrogen and methanogenesis are inconsistent. N additions either decreased, had no effect, or increased the rates of methanogenesis, possibly because of differences among studies in terms of the nitrogen form (NH_4_NO_3_, NO_3_^–^, NH_4_^+^, or urea), the amount of N added, the wetland community type, and the background N availability. For example, NH_4_NO_3_ significantly enhanced CH_4_ production in the soils of tidal *Suaeda japonica* marshes and bare tidal flats but did not significantly affect CH_4_ production in the soils of *Carex* and *Phragmites* marshes [53]. CH_4_ flux increased in coastal salt marshes when N was added in the form of slow-release urea [5]. In contrast, NH_4_^+^ additions decreased the rates of CH_4_ production in peatlands [15], but CH_4_ production in rice field soil was not affected by (NH_4_)_2_HPO_4_ [54]. High NO_3_^–^ inputs inhibited CH_4_ production in rice field soil [55], and NO_3_^–^ is thought to generally inhibit CH_4_ production in wetlands sediments [56]. In addition to the possible toxic effect of products of nitrate reduction (NO, NO_3_^−^ and N_2_O) on methanogens, nitrate reducers can outcompete methanogens for organic substrates [57]. Few studies have been published on the effects of N addition on other SOC mineralization pathways including Fe(III) and SO_4_^2–^ reduction, and the question about the response of soil C decomposition to globally rapid increases in reactive N is unsettled.

### 4.2 Effects of SLR and increases in inundation on SOC mineralization

We found that SLR inundation increase (+I) did not significantly change the rates of total organic C mineralization, Fe(III), SO_4_^2–^, or NO_3_^–^ reduction, or methanogenesis (Fig. 2). This may be because the Control group still remains normal flooding due to regular semi-diurnal tides, and the increase in flooding time has a minimal impact on the redox environment [31]. The original microbial community has already adapted to long-term flooding, thus resulting in a relatively small effect on bacterial abundance (Table 2). Furthermore, we found no significant changes in abundances of bacteria and electron acceptors of Fe(III), SO_4_^2–^, or NO_3_^–^ compared to the Control group (Table 1). Few papers have discussed the impact of changes in inundation on SOC loss, and most were in microcosm or laboratory incubation experiments. Increased inundation lowered CO_2_ emissions but raised CH_4_ emissions in coastal tidal wetlands [58], but lowered the rate of CO_2_ flux from a mesohaline mangrove peat soil [16]. In contrast, increased flooding did not affect soil respiration in field-based mesocosms in the absence of plants but dramatically increased respiration in the presence of plants [8]. In addition to the effect of duration, the pattern or hydroperiod is probably important. For example, forested wetland soils shifted from CH_4_ sinks to sources when experimentally pulsed with water [59].

The combination of N addition and increased inundation (+N+I) did not significantly change the rates of soil total organic C mineralization or the four organic C mineralization pathways (Fig. 2). This is because of dilution effect by inundation could lower levels of nutrients in pore water [60]. The results indicate that increases in SLR and inundation could offset the priming effects of N addition on the rates of soil total organic C mineralization, Fe(III), SO_4_^2–^, and NO_3_^–^ reduction, and methanogenesis in an estuarine tidal oligohaline marsh.

## 5 Conclusion

In this study, we used temporary weirs vegetated with *C. malaccensis* in a tidal oligohaline marsh to simulate the effects of SLR. The weirs allowed us to manipulate *in situ* inundation time to measure effects on SOC mineralization rates and pathways over two growing seasons. To some weirs, we added a mixture of NaNO_3_ plus NH_4_Cl, while others either served as controls or were treated with a combination of increased flooding plus nitrogen additions. The addition of N alone increased the rates of total SOC mineralization and soil microbial Fe(III), NO_3_^–^, and SO_4_^2–^ reduction but decreased the rate of methanogenesis. An increase in the duration of inundation did not change the rates of total SOC mineralization or the other microbial respiration pathways. Increased inundation offset the stimulatory effect of N addition on the rates of total SOC mineralization and Fe(III), SO_4_^2–^, and NO_3_^–^ reduction. The contributions of the Fe(III) and SO_4_^2–^ reduction pathways to total SOC mineralization increased with greater flooding, N enrichment, and in combination. The mechanisms by which N addition and flooding affect SOC mineralization involve Fe(III), SO_4_^2–^, and NO_3_^–^ reduction pathways as well as methanogenesis, which were positively correlated with Fe(III)/Fe(II) ratio and the abundance of *Geobacter, dsr*A, *nrf*A, and *mcr*A, BG and PHO.

The results of the present study indicated that the addition of nitrogen indirectly increased the DOC concentrations and the Fe(III)/Fe(II) ratio. Increased DOC presumably stimulated extracellular enzyme activity and anaerobic microbial respiration. Compared to the +N treatment, increased flood duration had little influence on either DOC or the Fe(III)/Fe(II) ratio. Therefore increased inundation did not significantly influence SOC mineralization. The present study had certain limitations. For example, we could not determine the redox potential for the increased SLR-inundation treatment. Tidal marshes can provide long-term, stable soil C storage. Increases in SLR-inundation might benefit soil C storage by attenuating the stimulatory influence of N loading enrichment on soil C loss in estuarine tidal marsh ecosystems. However, this effect is negated if increases in SLR result in the submergence and loss of existing coastal wetlands. Additionally, the methane reduction patterns (opposite to sulfate and iron reduction) are occurring because microbial communities are shifting towards more efficient terminal electron acceptors (TEAs).

## Data availability statement

The code and data necessary to reproduce the analyses are available at https://figshare.com/s/6f2e614de729019d189f.

## CRediT authorship contribution statement

**Chuan Tong:** Writing – review & editing, Writing – original draft, Formal analysis, Conceptualization. **Ji Tan:** Writing – review & editing, Investigation, Formal analysis. **Min Luo:** Writing – review & editing, Writing – original draft, Formal analysis. **Jiafang Huang:** Writing – review & editing, Investigation, Formal analysis. **Shuyao Xiao:** Writing – review & editing, Investigation, Formal analysis. **Baigui Liu:** Writing – review & editing, Investigation, Formal analysis. **James T. Morris:** Writing – review & editing, Writing – original draft, Formal analysis.

## Declaration of competing interest

The authors declare that they have no conflicts of interest in this work.

## Appendix Supplementary materials

Image, application 1
