# Supplementary material for: Inundation counteracts the promoting effect of nitrogen enrichment on soil organic carbon mineralization in a tidal marsh
Source: Fundam Res. 2024 Feb 5;6(1):301–12. doi: 10.1016/j.fmre.2024.01.013 (PMC12869760; doi:10.1016/j.fmre.2024.01.013)
Supplement: Supplementary file 1 [file mmc1.doc]

**[Supplementary Material]**

**Article type:** Primary Research Article

**Inundation counteracts the promoting effect of nitrogen addition on soil organic carbon mineralization in a tidal marsh**

Chuan Tong^a,b⁎^, Ji Tan^b^, Min Luo^c^, Jiafang Huang^a,b^, Shuyao Xiao^b^, Baigui Liu^b^, James T. Morris^d,**^

^a^Key Laboratory of Humid Sub-Tropical Eco-Geographical Processes (Ministry of Education), Fujian Normal University, Fuzhou 350007, China

^b^School of Geographical Sciences, Fujian Normal University, Fuzhou 350007, China

^c^College of Environment and Safety Engineering, Fuzhou University, Fuzhou 350108, China

^d^Belle Baruch Institute for Marine & Coastal Sciences, University of South Carolina, Columbia, 29208, USA

**Corresponding author information:**

*Chuan Tong

Telephone: 086-0591-87445659

E-mail: [tongch@fjnu.edu.cn](mailto:tongch@fjnu.edu.cn)

Fax: 086-0591-83465397

**James T. Morris

Telephone: 803-960-9351;

Email: morris@biol.sc.edu

Fax: 803-777-3935

**Caption:**

**Fig.** **S1** Schematic diagrams of passive weir. (a) Plan view showing the weir with four walls and two kinds of valves. Solid arrow direction and thickness indicate the direction and relative volume of water flow respectively. (b) Side view showing the installation of the weir, the arrangements of the litterbag and the water level recorder, and the details of the ball valve.

**Fig. S2** Comparison of the water levels in sea-level rise (SLR) treatment and Control treatment in 21-26 January, and 01-06 August 2019, respectively. Blue solid line: the water level in SLR treatment; red dotted line: the water level in Control treatment


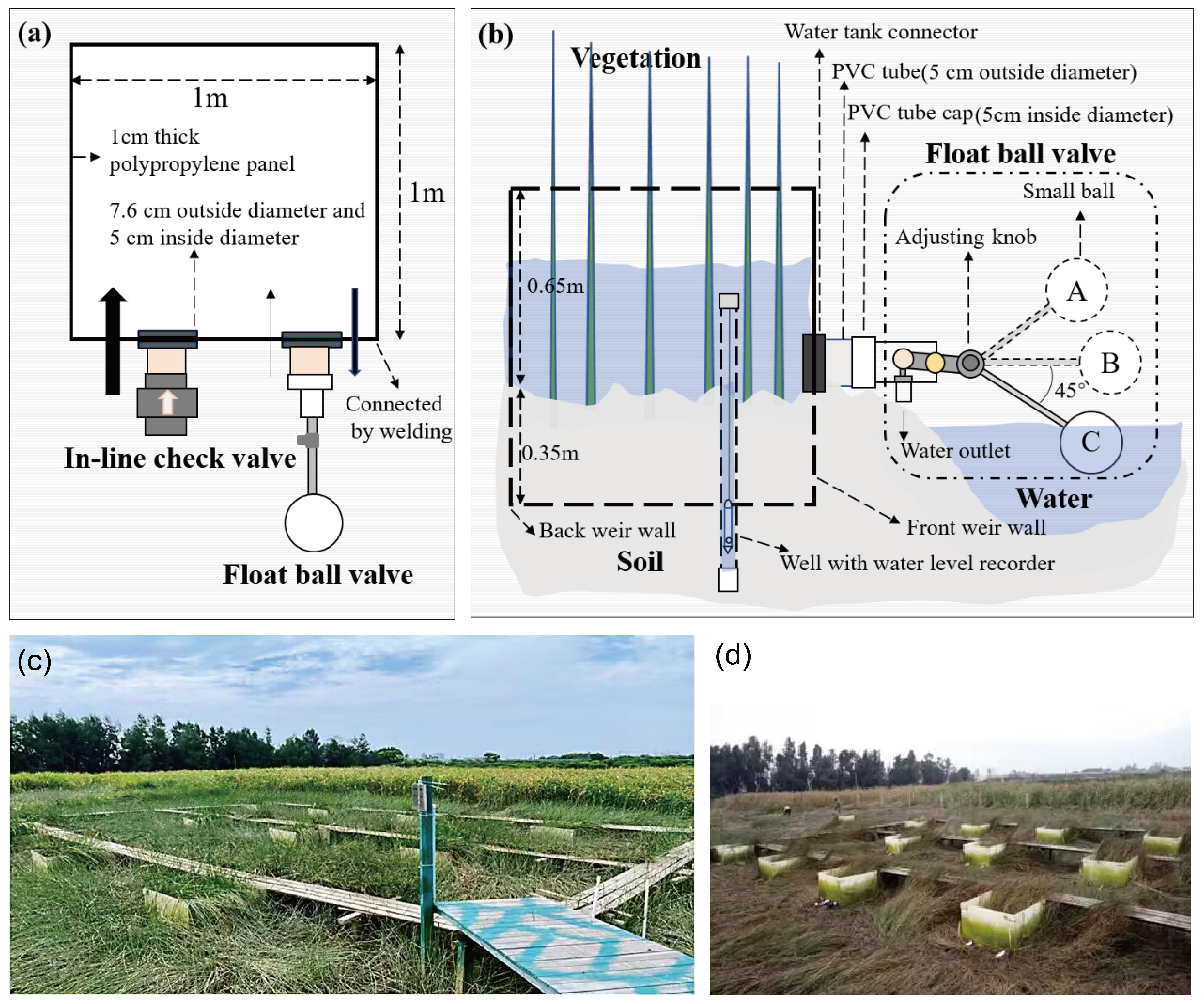


**Fig.** **S1** Schematic diagrams of passive weir. (a) Plan view showing the weir with four walls and two kinds of valves. Solid arrow direction and thickness indicate the direction and relative volume of water flow respectively. (b) Side view showing the installation of the weir, the arrangements of the litterbag and the water level recorder, and the details of the ball valve. (c–d) photos of the in-situ experiments.


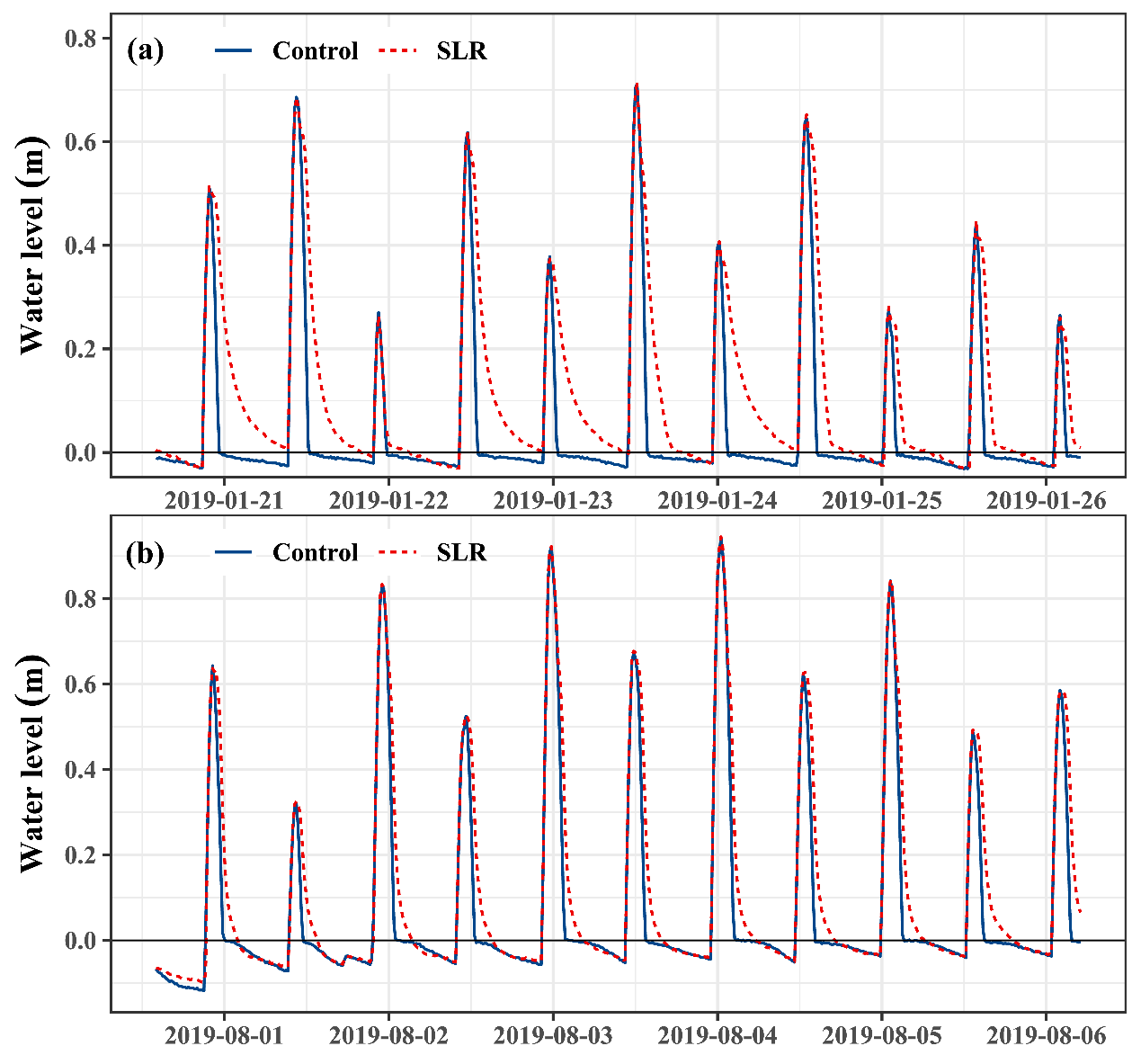


**Fig. S2** Comparison of the water levels in sea-level rise (SLR) treatment and Control treatment in 21-26 January, and 01-06 August 2019, respectively. Blue solid line: the water level in SLR treatment; red dotted line: the water level in Control treatment.
